# Optimization of nanopore sequencing for surveillance of antimicrobial resistance in low-resource settings

**DOI:** 10.3389/fpubh.2026.1755877

**Published:** 2026-02-24

**Authors:** Natasia R. Thornval, Niamh Lacy-Roberts, Ana Rita Rebelo, Pernille Nilsson, Joana Mourão, Christa Gibson, Henrik Hasman, Rene S. Hendriksen

**Affiliations:** 1 Research Group for Global Capacity Building, National Food Institute, Technical University of Denmark, Lyngby, Denmark; 2 National Reference Laboratory for Antimicrobial Resistance, Department of Bacteria, Parasites and Fungi, Statens Serum Institut, Copenhagen, Denmark

**Keywords:** antimicrobial resistance, bacteria, capacity building, glass, low-resource settings, nanopore sequencing, surveillance, WGS

## Abstract

Whole-genome sequencing (WGS) is emerging as a valuable tool for antimicrobial resistance (AMR) surveillance, yet implementation in low-resource settings remains limited by prohibitory costs and infrastructure constraints. Oxford Nanopore Technologies (ONT) offers portable sequencing platforms that can overcome these barriers, but optimal workflows for bacterial WGS are not fully standardized. We evaluated the impact of multiplexing level (12-, 24-, and 36-plex) and input DNA amounts (50 ng, 100 ng, and 200 ng) on sequencing performance using ONT’s Rapid Barcoding Kit v14 and R10.4.1 flow cells. Sequencing success was defined as assemblies with ≥30 × depth of coverage, complete MLST assignment, and full AMR gene detection. Across nine run configurations, sequencing success was highest for 12-plex runs (92–100% success) and 24-plex runs (79–82% success) when using ≤100 ng DNA input. 36-plex configurations and high DNA input markedly reduced performance (as low as 2.8% success). Lower DNA input (50 ng) did not compromise outcomes and mitigated negative effects of multiplexing. Cost analysis showed per-sample costs decreased with higher multiplexing, but excessive batching compromised data quality. These findings support practical ONT workflows for decentralized AMR surveillance, recommending ≤24 samples per flow cell and ≤100 ng DNA input to balance cost-effectiveness and sequencing success in low-resource laboratory settings.

## 1 Introduction

Antimicrobial resistance (AMR) is a major global health challenge. While whole-genome sequencing (WGS) adoption for routine surveillance is advancing in select high-income countries (1–3), implementation in low-and-middle income countries (LMICs) has been limited due to barriers such as high startup cost, infrastructure constraints, and shortages in bioinformatic capacity (1–5).

For resource-limited laboratories, Oxford Nanopore Technologies (ONT) MinION portable sequencing platform offers a promising alternative to sequencing platforms such as Illumina, or PacBio for that matter (6). The MinION’s low start-up equipment cost, minimal laboratory requirements, and compatibility with standard computing hardware make the MinION uniquely suitable for decentralized AMR surveillance and outbreak response (7–9). However, ONT-based workflows still require dedicated validation and harmonization for standardized bacterial WGS, reflecting a less established portfolio of protocols compared to Illumina (9–12). Current recommendations vary widely, with suggestions of up to 24 samples per flow cell, while others advise limiting multiplexing to 12–18 isolates (13–17). This lack of harmonization creates uncertainty for laboratories seeking to balance throughput, cost, and data quality.

This study was conducted as part of *‘SeqAfrica*’, a UK International Development Fleming Fund program, launched to establish and expand WGS based surveillance of AMR in the Sub-Saharan African region (18). The work was conducted in preparation for rolling out ONT-based workflows at decentralized laboratories, a strategy intended to democratize sequencing, reduce centralization, and improve geographical coverage of surveillance data.

Building upon our previous work optimizing DNA extraction for ONT-based AMR surveillance (19), this study evaluates the next step in the workflow: library preparation. Specifically, we assessed the impact of multiplexing level (12-, 24-, and 36-plex) and input DNA amounts (50 ng, 100 ng, and 200 ng) on sequencing performance using ONT’s Rapid Barcoding Kit v14 and R10.4.1 flow cells and evaluated the sequencing success, defined as assemblies with ≥30 × depth of coverage, complete MLST assignment, and full AMR gene detection, to identify practical configurations that maximize sequencing success while remaining feasible for low-resource laboratories.

## 2 Materials and methods

### 2.1 Bacterial strains and DNA extraction

Four strains representing both Gram-positive and Gram-negative bacterial species were selected for this study: *Campylobacter coli* GPT20-006*, Escherichia coli* GPT22-004, *Enterococcus faecalis* GPT22-006, and *Staphylococcus hominis* NT-2025, as described in our previous study (19). These strains had previously been sequenced with PacBio or hybrid assembly with ONT and Illumina (hybrid assembly), resulting in complete circular reference genomes as described in Table 1. Genomic DNA (gDNA) was extracted using the DNeasy Blood and Tissue spin-columns kit (Qiagen, Hilden, Germany) according to the manufacturer’s protocol with modifications: all post-lysis vortexing steps were replaced with flicking the tube and pipetting gently up-and-down to mix, and 15 mg/mL lysostaphin was added for lysis of the *S. hominis* strain.

**Table 1:** Overview of strains in this study.

Strain name	Species	NCBI accession number	Sequence type (ST)	Total base pairs (bp)	GC % content	No. of Plasmids	ARG
GENOMIC22-004	*Escherichia coli*	GCA_029094485	410	5,164,118	50.5	6	*rmtC*, *aac(6′)-lb3*, *bla*_CMY-6_, *bla*_NDM-1_, *bla*_OXA-181_, *qnrS1*, *sul1*
GENOMIC20-006	*Campylobacter coli*	GCA_949361535	3,336	1,814,825	31.5	1	*aadE-Cc, bla*_OXA-193_, tet(O)
GENOMIC22-006	*Enterococcus faecalis*	GCA_029167565	6	3,407,461	37.5	3	*aac(6′)-aph(2″), lsa(A), erm(B), cat(pC221), tet(M), VanHBX*
NT-2025	*Staphylococcus hominis*	JBRFSD000000000	223	2,337,290	31.5	4	*aadD, blaZ, fusB, tet(k), bleO*

### 2.2 Library preparation and whole genome sequencing

To evaluate the effect of multiplexing level and DNA input concentration, nine run configurations were generated by combining three DNA concentrations (5 ng/μl, 10 ng/μl, and 20 ng/μl) with three multiplex levels (12-, 24-, and 36-plex). For each run configuration, 10 μL of gDNA was used per sample, corresponding to final input amounts of 50 ng, 100 ng, and 200 ng measured with Qubit 4 Fluorometer (Invitrogen, Thermo Fisher Scientific, Waltham, MA, USA) using the Qubit High Sensitivity Quantification Kit (Invitrogen, Thermo Fisher Scientific), except for concentrations >120 ng/μl where the Invitrogen Qubit Broad Range Quantification kit (Invitrogen, Thermo Fisher Scientific) was used according to the manufacturer’s protocol.

Each strain was represented across all three DNA concentrations and included in multiple technical replicates within each multiplexing level. Library preparation was performed using the Rapid Barcoding kit v14 (SQK-RBK114.96).

The prepared libraries were loaded onto R10.4.1 flow cells (FLO-MIN114) with more than 800 active pores pre-sequencing. Flow cells were sequenced using ONT’s MinION or GridION sequencing platforms with the MinKNOW v23.11.4 software. Basecalling was performed using the super-accurate basecalling (SUP) model, and reads were filtered based on a minimum Phred quality score (Q-score) > 10 and read length > 400 bp thresholds.

### 2.3 Bioinformatic and statistical analysis

Genomes were assembled with Flye v2.9.1 (20) using default settings, and per-sample read metrics were generated with NanoStat v1.6.0 (21). As in our previous study (19), MLST profiles and AMR genes (ARG) were identified for the reference genomes and assemblies using our previously described in-house pipeline (22), which employs MLST v2.0 (22) with 100% identity and 100% coverage, and ResFinder v4.2.3 (database version 2.3.2) (23, 24) with ≥ 95% identity and ≥ 95% coverage.

R statistical software v4.4.2 (25) in RStudio (26) was used for all statistical analyses and all plots were visualized using the ggplot2 package (27). To assess the influence of the run configuration on the WGS performance, Firth-penalized logistic regression was conducted using the logistf package (28). The model evaluated the effects of multiplexing level and input DNA concentration on four binary outcomes: (a) achieving ≥ 30 × sequencing depth, (b) complete MLST locus detection, (c) full ARG detection, and (d) overall WGS success, defined as meeting all three criteria. The model incorporated an interaction term between multiplexing level and DNA input concentration and was adjusted for bacterial strain to account for strain-specific variability. The model fit was assessed using likelihood ratio test, and odds ratios (OR) with 95% confidence intervals (CI) reported relative to the reference condition (12-plex × 100 ng input DNA). Statistical significance was defined as *p* < 0.05. For each run configuration, the binary outcomes were summarized as *n*/*N* (%), where *n* is the number of samples meeting the criterion, and *N* is the number evaluated. The workflow from sequence data generation to analysis and how WGS success was defined can be found in Supplementary Figure S1.

### 2.4 Cost estimation

All cost estimates were reported in Euros (€), exclusive of value added tax (VAT). Costs associated with auxiliary equipment and personnel were excluded from the cost analysis. The cost per sequencing run was calculated based on prices from Danish suppliers as of January 2025 and reported both by including and excluding DNA extraction, covering the process from library preparation to finalized WGS data. Per-sample costs were calculated for each multiplexing level. Costs for ONT flow cells were derived from single-unit purchase prices. The library preparation was estimated using the Rapid Barcoding kit 96 v14 and included additional consumables not supplied with the kit (i.e., pipette tips, tubes, and chemicals). Cost per sample was determined for each specific multiplexing level.

## 3 Results

### 3.1 Sequencing output across run configurations

**Figure 1:**
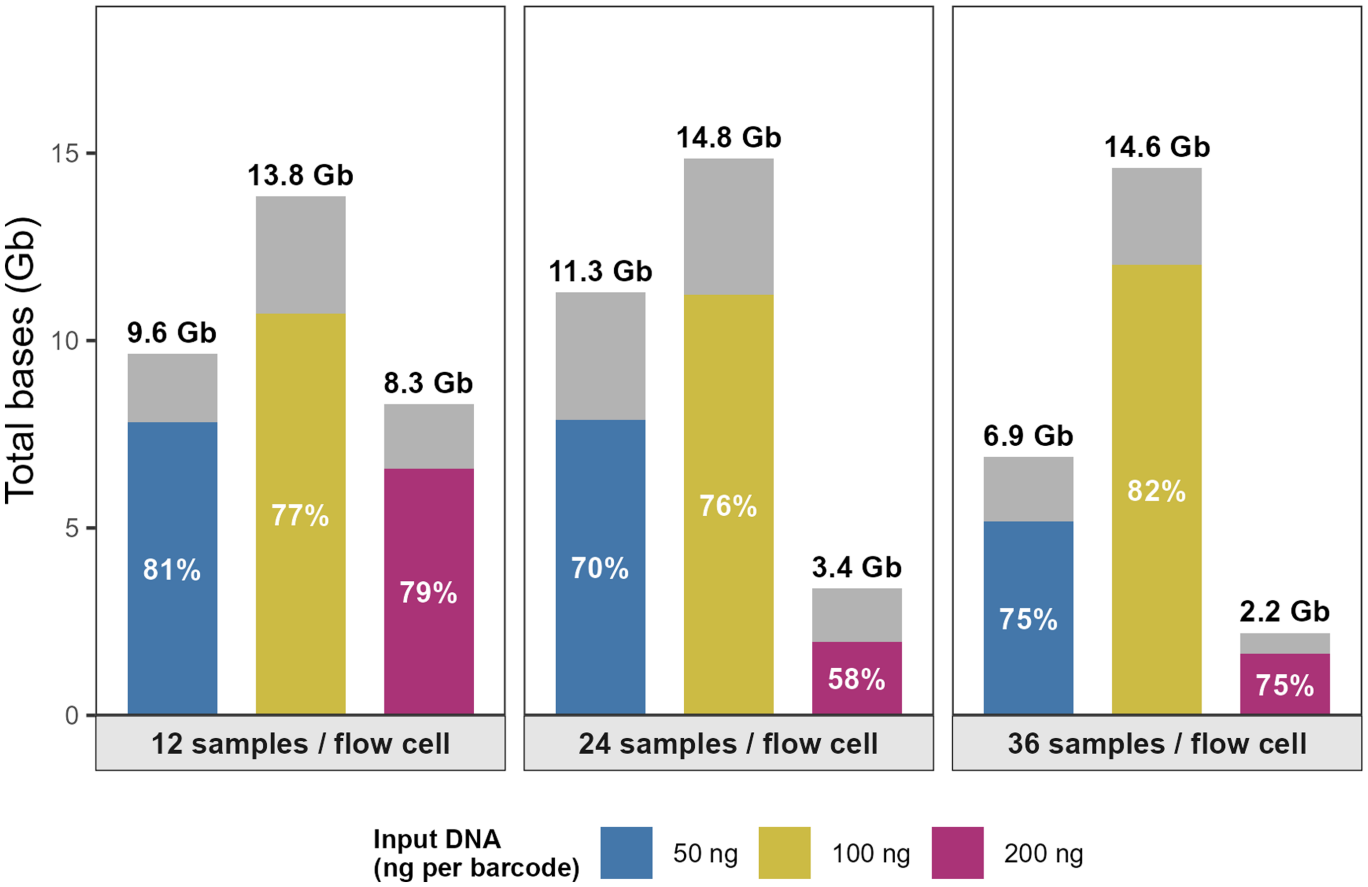
Sequencing yield and proportion of high-quality data across multiplexing levels and input DNA concentrations. Stacked bar plots show the total bases generated per flow cell (Gb) for each sequencing run configuration, defined by multiplexing configurations: 12, 24, and 36 samples per flow cell and input DNA concentrations: 50 ng/μl (blue), 100 ng/L (yellow), and 200 ng/L (purple). The colored lower segment represents basecalled data passing quality filtering (Q-score ≥ 10, read length ≥ 400 bp), while the grey upper segment represents basecalled reads failing QC or reads that could not be assigned to a barcode. Total yield per run (Gb) is indicated above each bar, and the percentage of bases passing QC is shown within the colored segment. Created with BioRender.com.

The ONT runs yielded between 2.2 and 14.61 gigabases (Gb) of data with a median output of 9.65 Gb. The highest data outputs were observed in the three runs using 100 ng input DNA with the 12-plex, 24-plex, and 36-plex configurations, which generated 13.85 Gb, 14.61 Gb, and 13.06 Gb, respectively, with 64.80 to 72.05% of reads passing quality thresholds of Q-score ≥ 10 and read length ≥ 400 bp, respectively. In comparison, the 12-plex and 24-plex runs using 50 ng input DNA produced 9.65 Gb and 11.29 Gb, respectively. Conversely, the 24-plex and 36-plex runs with 200 ng input DNA yielded less data, 3.38 Gb and 2.20 Gb, respectively, although 71.93 to 75.56% of reads passed quality filtering (Figure 1).

### 3.2 Effect of run configurations on sequencing performance

#### 3.2.1 Model performance and explanatory power

Firth logistic regression models showed a significant difference between run configurations and all binary outcomes assessed. These included sequencing depth ≥30 × and overall sequencing success (likelihood ratio test: χ^2^(11) = 141.0, R^2^ = 0.67, *p* = 1.1 × 10^−23^), detection of full MLST (χ^2^(11) = 96.9, R^2^ = 0.60, *p* = 7.4 × 10^−16^), and detection of all ARG (χ^2^(11) = 49.6, R^2^ = 0.47, *p* = 7.4 × 10^−7^). All samples with a sequencing depth ≥30 × achieved overall WGS success, resulting in identical model fit for these two outcomes.

#### 3.2.2 Effect of multiplexing level and DNA input

**Figure 2:**
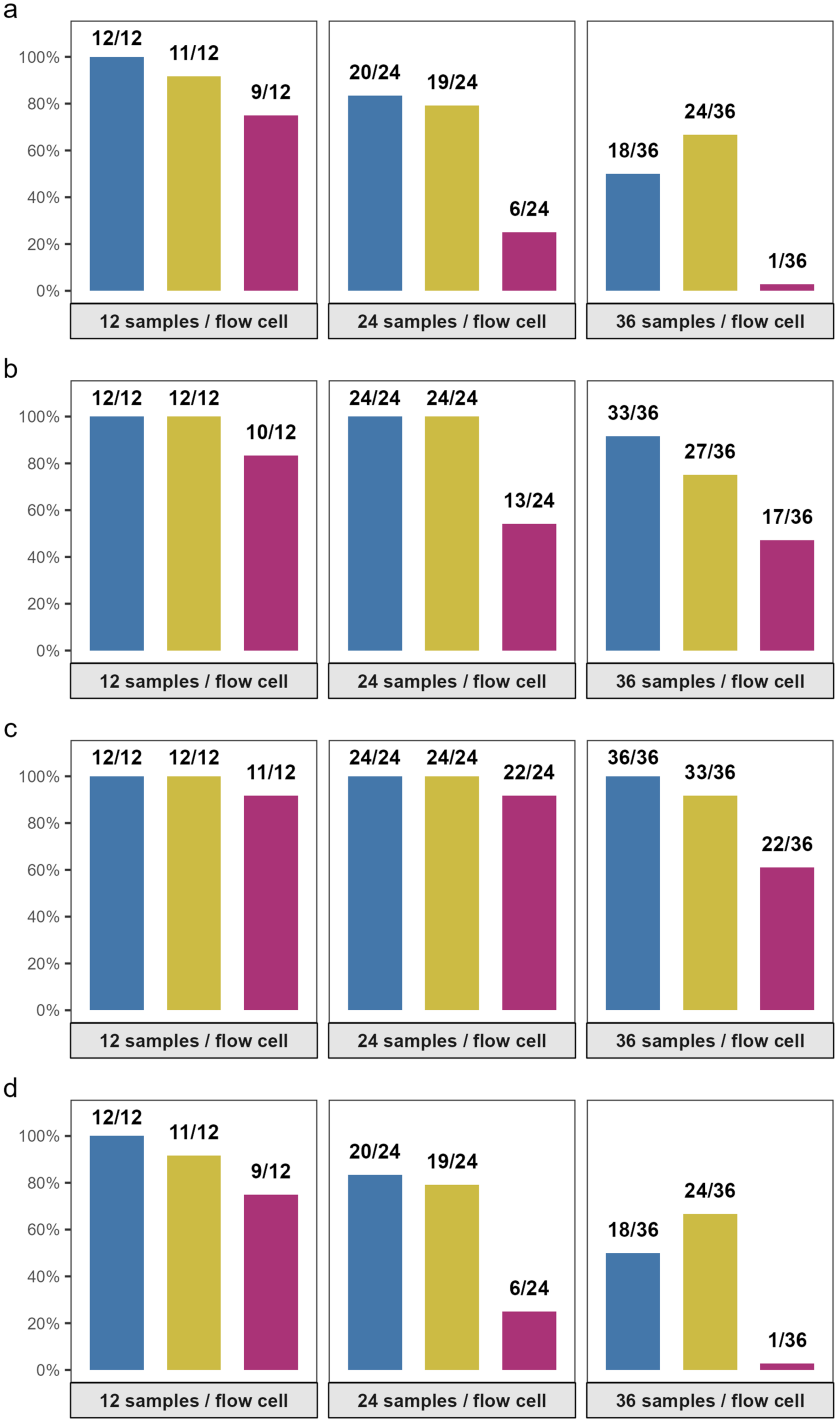
Effect of multiplexing level and input DNA on sequencing outcomes. Bar plots show the proportion of samples with successful binary outcomes across different multiplexing configurations: 12, 24, and 36 samples per flow cell and input DNA concentrations: 50 ng/μL (blue), 100 ng/μL (yellow), and 200 ng/μL (purple). Panels display proportion of samples with **(a)** ≥ 30 × sequencing depth, **(b)** all MLST loci detected, **(c)** all AMG detected, and **(d)** overall sequencing success, defined as meeting all three criteria. The number of success samples (*n*) out of the total analyzed (*N*) is indicated above each bar. Created with BioRender.com.

At the reference condition (12-plex × 100 ng input DNA), 91.67% of samples (11/12) achieved ≥30 × depth and met the complete WGS success criteria, which included complete detection of MLST and ARGs. Increasing the multiplexing level at the same input concentration reduced the odds of WGS success, although not significantly: 24-plex × 100 ng yielded 79.17% success (19/24, OR = 0.36, 95% CI 0.03–2.7, *p* = 0.33), and 36-plex × 100 ng yielded 66.67% success (24/36, OR = 0.154, 95% CI 0.01–1.01, *p* = 0.05) (Figures 2a–d, Supplementary Table S2).

In contrast, reducing the input DNA to 50 ng did not negatively affect WGS performance. The 12-plex × 50 ng run configuration achieved 100% success (12/12, OR = 3.76, 95% CI 0.15–614.95, *p* = 0.43), and the 24-plex configuration maintained high success (20/24, 83.33%, OR = 0.37, 95% CI 0–13.29, *p* = 0.60). However, even at low DNA input, the high multiplex level (36-plex × 50 ng) showed reduced performance, yielding only 50.00% success (18/36, OR = 3.76, 95% CI 0.15–614.95, *p* = 0.43).

The negative impact of increased multiplex-level was most pronounced at high DNA input. The 36-plex × 200 ng configuration achieved only 2.8% WGS success (1/36), with a significant reduction in odds (OR = 0.02, 95% CI 0.0008–0.58, *p* = 0.024). A similar trend (25%) was observed for the 24-plex × 200 ng configuration (6/24, OR = 0.14, 95% CI 0.01–2.82, *p* = 0.19), while the 12-plex × 200 ng run showed moderate success (9/12, 75.00%, OR = 0.25, 95% CI 0.02–2.52, *p* = 0.25) (Figure 2d, Supplementary Table S2).

#### 3.2.3 MLST and ARG detection

Across all models, the negative effect of high multiplexing was most evident for sequencing depth and overall WGS success. In contrast, MLST and ARG detection remained relatively stable. Complete MLST assignment (100%) was achieved in all run configurations except those with high multiplex level (36-plex) and/or high DNA input (200 ng). ARG detection exceeded 90% across most configurations, except for the 36-plex × 200 ng run, which showed reduced detection (22/36, 61.11%) (Figures 2b,c).

#### 3.2.4 Strain specific performance

Among the four bacterial strains, *C. coli* exhibited the greatest decline from the reference condition in WGS success, accounting for 40.62% of all failed WGS samples (39/96). WGS success rates for the *C. coli* strain decreased markedly with increasing multiplex level, from complete success at 12-plex × 50 ng (3/3, 100%) to ≤33.33% success across all 24-plex configurations, and ranged from 0.00–66.67% at 36-plex, with no successful WGS at 36-plex using 100 ng or 200 ng input. The *E. faecalis* strain demonstrated the highest robustness, accounting for only 11.46% of all failures (11/96) and achieving 100.00% success across all 12-plex and 24-plex configurations. Reduced performance was observed only at 36-plex × 200 ng, where no successful WGS was achieved, while high success was retained at 36-plex × 50 ng (7/9, 77.78%). The *E. coli* and *S. hominis* strains showed intermediate performance, each contributing 23.96% of all failed samples. For both strains reduced success was particularly evident at high DNA input (200 ng), especially in 24-plex and 36-plex configurations, where success rates declined to ≤ 11.11% (Supplementary Table S3).

### 3.3 Cost analysis by multiplexing level

The cost per ONT sequencing run, including DNA extraction, ranged from €791.35 to €803.23. Cost per sample varied substantially depending on multiplexing level: 12-plex runs were the most expensive at €65.95 per sample (or €72.05 when including DNA extraction), followed by 24-plex at €33.22 (€39.32 with extraction), and 36-plex €22.31 (€28.41 with extraction). Thus, increasing multiplexing from 12 to 36 samples reduced the per-sample cost by approximately 60%, nearly a threefold difference (Table 2). Across all run configurations, flow cell expenses accounted for the largest proportion of the cost, ranging from 68.44 to 80.97% of the total per-sample cost.

**Table 2:** Cost associated with multiplexing level.

Level of multiplexing	Cost per sample for WGS ^1^	Cost per sample for WGS incl. DNA extraction^1^
12-plex	€65.95	€72.05
24-plex	€33.22	€39.32
36-plex	€22.31	€28.41

1 The cost per sample is calculated in Euro (€) per sample if bought in Denmark and included costs of reagents not included in the SQK-RBK114.96 kit (i.e., pipette tips, tubes, and chemicals). All prices were pre-tax totals as of Jan 2025. The startup cost for auxiliary equipment and other associated running cost were not included in cost per sample.

## 4 Discussion

ONT’s recommended input DNA for the SQK-RBK-114 kit changed from 50 ng (RBK_9126_v110_revO_24Mar2021) to 200 ng (RBK_9176_v114_revM_27Nov2022 and subsequent protocols) on the 27th of November 2022. Considering these changes, we evaluated whether the revised recommendations improved WGS performance. Our study compared three multiplexing levels (12-plex, 24-plex, and 36-plex) and three input DNA amounts (50 ng, 100 ng, and 200 ng) to identify configurations that balance sequencing success, throughput, and cost-effectiveness for surveillance of AMR in low-resource settings.

### 4.1 Impact of run configurations on sequencing performance

Our study indicates the interaction between multiplex level and input DNA influenced WGS success significantly. The results show a clear interaction between multiplexing level and DNA input. Runs with 12-plex and 24-plex configurations using ≤100 ng DNA consistently achieved high sequencing depth, accurate MLST assignment, and robust ARG detection. In contrast, 36-plex runs showed reduced success, particularly when combined with 200 ng input DNA, where overall WGS success dropped to 2.8%. The 24-plex and 36-plex runs with 200 ng input DNA yielded a lower data output (≤3.4 Gb data) than the other run configurations in the study (≥6.9 Gb data) (Figure 1). Interestingly, reducing DNA input to 50 ng did not negatively affect performance; in fact, 12-plex × 50 ng achieved 100% success, and 24-plex × 50 ng maintained >80% success (Figures 2a–d).

These findings suggest that lower DNA loading can mitigate some negative effects of high multiplexing, whereas excessive DNA input combined with large batch sizes significantly compromises sequencing efficiency. This supports previous protocols recommending 100 ng input DNA for multiplexing >4 samples (29), however, does not align with ONT’s own protocols.

Our findings align partially with other studies and protocols. Landman et al. (17) recommends multiplexing maximum 16–18 bacterial samples for AMR surveillance using the ONT SQK-RBK-114 library preparation kit and R10.4.1 flow cells, whereas other studies have successfully multiplexed 10–12 bacterial samples for species such as *Clostridioides difficile* and *Salmonella* (13, 30). ONT’s own NO-MISS protocol (ISO_9205_v114_revG_09May2025) describes a flexible range of multiplexing 4–24 bacterial isolates per flow cell. The reduced WGS success observed in our 36-plex runs supports these guidelines, indicating that multiplexing beyond 24 samples may compromise data quality, particularly when paired with high DNA input.

### 4.2 Technical considerations

The 24-plex and 36-plex runs with 200 ng input DNA yielded a lower data output (≤3.4 Gb data) than the other run configurations in the study (≥6.9 Gb data) and a reduced WGS performance was observed with high multiplexing level (36-plex) and input DNA (200 ng) (Figures 1, 2).

It is important to note that the DNA extraction method and library preparation kit used in this study were deliberately selected to maintain feasibility in low-resource laboratory settings. The DNeasy Blood & Tissue spin-columns kit was selected because it is widely available in LMICs, and is cost-efficient, time-efficient, and generally robust, despite not being specifically optimized for high-molecular weight (HMW) DNA. The protocol does not include any RNA removal steps or size selection. Incorporating such steps would require additional reagents or separate kits, thereby increasing both cost and logistical burden and reducing overall cost-effectiveness (19). Similarly, the SQK-RBK114 kit employs a transposase-based fragmentation and barcoding step without purification to remove short DNA fragments, instead prioritizing simplicity and speed over long read lengths and yield. In contrast, ONT’s ligation-based library preparation kits incorporate multiple purification steps that remove short fragments, thereby enriching for HMW DNA and generally yielding higher total data output (9, 15, 31). However, small fragments will be preferentially sequenced on the ONT platform due to a higher translocation speed than HMW (32). The Nanopores wear down faster with small molecular wight DNA, thereby limiting the total amount of generated Gb yield (33). These effects could accumulate with increased input mass (both from level of multiplexing and input DNA quantity), thereby causing the reduced data yield observed for 24-plex and 36-plex runs with 200 ng input DNA. An reduced performance across run configurations may also reflect inhibitory effect from EDTA in the DNeasy Blood & Tissue elution buffer (34). Although ONT reports significant performance reduction of rapid sequencing kit at 5 mM of EDTA, even the 0.5 mM concentration in the buffer may have contributed to reduced efficiency when compounded with other factors at high multiplexing (35). A different elution buffer without EDTA could have avoided this potentially inhibitory effect, however that would have required procurement of additional reagents not included in the commercial kit. Any additional reagents were avoided, if possible, in the study to maintain feasibility in LMICs.

The reduced performance for high multiplexing and input DNA can in large part likely be attributed to the lack of additional purification steps in the DNA extraction and library preparation.

### 4.3 Robustness for GLASS and FAO priority pathogens

The four strains included in our study had varied genome sizes (1.8–5.1 Mb) (Table 1). If applying this workflow to WGS runs containing only large genomes (e.g., *Enterobacterales*), a lower sequencing depth would be generated per Gb. This is a consideration when designing a workflow usable for all World Health Organization (WHO) Global Antimicrobial Resistance and Use Surveillance System (GLASS) and the Food and Agriculture Organization of the United Nations (FAO) priority pathogens and could indicate that multiplex levels above 24 samples may be unsuitable.

Despite libraries being normalized and N50 being comparable across the four strains, the *Campylobacter* strain yielded fewer WGS successes, primarily due to low sequencing depth (Supplementary Table S3). This underrepresentation is unlikely to be attributed to the species’ low GC content, as the *C. coli*, *E. faecalis,* and *S. hominis* strains all exhibited similar levels yet achieved high WGS success. DNA fragmentation and loading bias have nevertheless been reported as challenges for *Campylobacter* due to its low GC content (36). Furthermore, genome size is an unlikely cause as the strain’s smaller genome size (~1.8 Mb) should theoretically correspond to a higher number of genome copies per ng/μl (Table 1). Instead, it might reflect strain-specific differences in transposase barcoding efficiency or genome copy-number effects, potentially compounded by barcode imbalance typical of high multiplexed ONT runs as seen in other studies (17, 37). ONT’s SQK-RBK kit uses the transposase-mediated fragmentation and adapter tagging (31). The lower yield for *Campylobacter* may reflect reduced transposase tagging efficiency, consistent with known reduction in sequencing performance associated with DNA composition, secondary structure, and integrity in transposase-based barcoding chemistries (2, 38, 39).

While the study included Gram-positive and Gram-negative strains with varied genome sizes, broader GLASS panels will include more extreme GC contents, where composition and kit-associated biases may exacerbate sequencing depth inequities in high-multiplex runs, highlighting the need for conservative depth targets (40). Many GLASS priority pathogens, particularly *Enterobacterales*, frequently carry ARG on plasmids and other mobile genetic elements that require sufficient depth for contextualization (41, 42). Our findings support low (12-plex) to moderate (24-plex) multiplexing strategies for GLASS implementation. Further, these findings highlight that laboratory workflow optimization is critical to ensure that genomic surveillance data remains interpretable and comparable across organisms and sequencing sites, particularly in decentralized surveillance settings, aligning with published implementation models and expert recommendations (3, 43).

### 4.4 Cost effectiveness in low-resource settings

Cost, infrastructure, and supply chain challenges remain the most significant barriers to WGS implementation in African laboratories (5, 44, 45). Our analysis focused exclusively on consumables (flow cells, library preparation kits, and DNA extraction) and excluded personnel, equipment, and infrastructure, as existing costing frameworks emphasise that consumables and reagents, rather than equipment purchase, are dominant recurring cost drivers for sustainable WGS implementation in LMICs (46, 47). Within this scope, ONT WGS using the DNeasy Blood & Tissue kit and SQK-RBK-114.96 library prep kit costs €865 to €1,023 per sequence run, with per-sample cost strongly influenced by multiplexing level: €72.05 for 12-plex, €39.32 for 24-plex, and €28.41 for 36-plex (Table 2). Flow cells accounted for approximately 68 to 81% of the total cost per isolate. African cost-analysis similarly reports even higher proportions (12-plex: ~86–91%, 24-plex: ~75–82%, and 36-plex: 67–76%) for similar workflows, likely due to transport and warranty cost for the flow cells (48).

However, run configurations that reduce per-sample cost at the expense of sequencing success may increase overall programme cost through repeat sequencing and delayed reporting, causing loss of actionable surveillance data. These risks are particular significant in settings such as Sub-Saharan Africa, where supply chains are characterized by long lead times and cold-chain constraints (49).

While increasing multiplex level reduces the per-sample costs, Elton et al. (50) noted that the time to results may be increased at laboratories that do not process a high number of samples, as these would need a longer period to collect enough samples to sequence. A possible mitigation strategy may be to wash and reuse the flow cells to increase cost-effectiveness whilst not compromising turnaround time or buying flow cells in bulk, which can lower costs up to ~25% (from €761.68 to €571.26 in Sudan, as of November 2025), though warranty periods, expirations dates, and procurement challenges remain significant (5, 45, 51, 52). These findings highlight that decentralized sequencing strategies must be planned with realistic expectations regarding sample throughput, turnaround time, and reagent availability, as excessive multiplexing may reduce data quality without delivering sustainable cost savings.

### 4.5 Practical implications and recommendations

Overall, our findings indicate that 12-plex runs are unlikely to be cost-effective for routine implementation in low-resource settings, where affordability is particularly critical (48). Multiplexing 24 samples using the SQK-RBK114 kit with low to moderate DNA input (50–100 ng) offers the most practical balance between sequencing success and cost for decentralized laboratories. These results highlight the importance of optimizing both multiplexing level and DNA input to ensure reliable ONT WGS outcomes. The findings support decentralized AMR surveillance strategies and align with global initiatives such as GLASS. Future studies should validate these recommendations across diverse bacterial species and explore additional cost-saving measures, including washing of flow cell for reuse and alternative library preparation kits, to enhance scalability and sustainability.

### 4.6 Study limitations

Although sequencing success declined from 91.7% at 12-plex × 100 ng to 66.7% at 36-plex × 100 ng, this difference was not statistically significant (OR = 0.15, 95% CI 0.01–1.01, *p* = 0.052). The lack of significance likely reflects limited sample sizes rather than absence of a biological effect. A stronger and statistically significant decline was observed when both multiplexing and input DNA were increased (36-plex × 200 ng; OR = 0.02, 95% CI 0.0008–0.58, *p* = 0.024), reinforcing that overloading flow cells substantially reduces sequencing performance.

The study used Rapid Barcoding Kit v14 and R10.4.1 flow cells. Use of older chemistries or alternative library preparation kits may yield different optimal configurations. Additionally, inclusion of only four bacterial strains may limit its applicability to broader AMR surveillance efforts, as it does not encompass the full spectrum of species typically encountered.

To further evaluate the applications of these findings, SeqAfrica has initiated multicenter implementation studies, including a pilot comparison of decentralized ONT WGS, using the workflow optimized here, with centralized Illumina sequencing for *Salmonella* as a model pathogen in South Africa, alongside One Health sentinel site activities in Ghana evaluating a broader panel of local priority pathogens in the GLASS bracket. Assessment using primary isolates from clinical and One Health surveillance contexts will be essential to evaluate robustness under routine surveillance conditions. Positioned as a foundational optimization study, this work provides a framework to inform subsequent implementation studies and capacity-building efforts for decentralized AMR genomic surveillance.

## Data Availability

The data is available in the National Center for Biotechnology Information (NCBI) Sequence Read Archive (SRA) under BioProject PRJNA1364846.
