# Supplementary material for: Optimization of nanopore sequencing for surveillance of antimicrobial resistance in low-resource settings
Source: Front Public Health. 2026 Feb 24;14:1755877. doi: 10.3389/fpubh.2026.1755877 (PMC12971681; doi:10.3389/fpubh.2026.1755877)
Supplement: Supplementary file 1 [file Supplementary_file_1.pdf]

Supplementary Material

Supplementary Figures and Tables

Supplementary Table S1: Sample overview		
**Accession**	**Title**	**BioSample**
SRR36035063	Campylobacter coli, DBT kit, 12-plex and 100 ng DNA, replicate 1	SAMN53262601
SRR36035062	Campylobacter coli, DBT kit, 12-plex and 100 ng DNA, replicate 2	SAMN53262601
SRR36035059	Campylobacter coli, DBT kit, 12-plex and 100 ng DNA, replicate 3	SAMN53262601
SRR36035058	Escherchia coli, DBT kit, 12-plex and 100 ng DNA, replicate 1	SAMN53262602
SRR36035057	Escherchia coli, DBT kit, 12-plex and 100 ng DNA, replicate 2	SAMN53262602
SRR36035056	Escherchia coli, DBT kit, 12-plex and 100 ng DNA, replicate 3	SAMN53262602
SRR36035055	Enterococcus faecalis, DBT kit, 12-plex and 100 ng DNA, replicate 1	SAMN53262603
SRR36035054	Enterococcus faecalis, DBT kit, 12-plex and 100 ng DNA, replicate 2	SAMN53262603
SRR36035053	Enterococcus faecalis, DBT kit, 12-plex and 100 ng DNA, replicate 3	SAMN53262603
SRR36035052	Staphylococcus faecalis, DBT kit, 12-plex and 100 ng DNA, replicate 1	SAMN53262604
SRR36035061	Staphylococcus faecalis, DBT kit, 12-plex and 100 ng DNA, replicate 2	SAMN53262604
SRR36035060	Staphylococcus faecalis, DBT kit, 12-plex and 100 ng DNA, replicate 3	SAMN53262604
SRR36036008	Campylobacter coli, DBT kit, 24-plex and 100 ng DNA, replicate 1	SAMN53262601
SRR36036007	Campylobacter coli, DBT kit, 24-plex and 100 ng DNA, replicate 2	SAMN53262601
SRR36035996	Campylobacter coli, DBT kit, 24-plex and 100 ng DNA, replicate 3	SAMN53262601
SRR36035991	Campylobacter coli, DBT kit, 24-plex and 100 ng DNA, replicate 4	SAMN53262601
SRR36035990	Campylobacter coli, DBT kit, 24-plex and 100 ng DNA, replicate 5	SAMN53262601
SRR36035989	Campylobacter coli, DBT kit, 24-plex and 100 ng DNA, replicate 6	SAMN53262601
SRR36035988	Escherchia coli, DBT kit, 24-plex and 100 ng DNA, replicate 1	SAMN53262602
SRR36035987	Escherchia coli, DBT kit, 24-plex and 100 ng DNA, replicate 2	SAMN53262602
SRR36035986	Escherchia coli, DBT kit, 24-plex and 100 ng DNA, replicate 3	SAMN53262602
SRR36035985	Escherchia coli, DBT kit, 24-plex and 100 ng DNA, replicate 4	SAMN53262602
SRR36036006	Escherchia coli, DBT kit, 24-plex and 100 ng DNA, replicate 5	SAMN53262602
SRR36036005	Escherchia coli, DBT kit, 24-plex and 100 ng DNA, replicate 6	SAMN53262602
SRR36036004	Enterococcus faecalis, DBT kit, 24-plex and 100 ng DNA, replicate 1	SAMN53262603
SRR36036003	Enterococcus faecalis, DBT kit, 24-plex and 100 ng DNA, replicate 2	SAMN53262603
SRR36036002	Enterococcus faecalis, DBT kit, 24-plex and 100 ng DNA, replicate 3	SAMN53262603
SRR36036001	Enterococcus faecalis, DBT kit, 24-plex and 100 ng DNA, replicate 4	SAMN53262603
SRR36036000	Enterococcus faecalis, DBT kit, 24-plex and 100 ng DNA, replicate 5	SAMN53262603
SRR36035999	Enterococcus faecalis, DBT kit, 24-plex and 100 ng DNA, replicate 6	SAMN53262603
SRR36035998	Staphylococcus faecalis, DBT kit, 24-plex and 100 ng DNA, replicate 1	SAMN53262604
SRR36035997	Staphylococcus faecalis, DBT kit, 24-plex and 100 ng DNA, replicate 2	SAMN53262604
SRR36035995	Staphylococcus faecalis, DBT kit, 24-plex and 100 ng DNA, replicate 3	SAMN53262604
SRR36035994	Staphylococcus faecalis, DBT kit, 24-plex and 100 ng DNA, replicate 4	SAMN53262604

SRR36035993	Staphylococcus faecalis, DBT kit, 24-plex and 100 ng DNA, replicate 5	SAMN53262604
SRR36035992	Staphylococcus faecalis, DBT kit, 24-plex and 100 ng DNA, replicate 6	SAMN53262604
SRR36068010	Campylobacter coli, DBT kit, 36-plex and 100 ng DNA, replicate 1	SAMN53262601
SRR36068009	Campylobacter coli, DBT kit, 36-plex and 100 ng DNA, replicate 2	SAMN53262601
SRR36067998	Campylobacter coli, DBT kit, 36-plex and 100 ng DNA, replicate 3	SAMN53262601
SRR36067987	Campylobacter coli, DBT kit, 36-plex and 100 ng DNA, replicate 4	SAMN53262601
SRR36067980	Campylobacter coli, DBT kit, 36-plex and 100 ng DNA, replicate 5	SAMN53262601
SRR36067979	Campylobacter coli, DBT kit, 36-plex and 100 ng DNA, replicate 6	SAMN53262601
SRR36067978	Campylobacter coli, DBT kit, 36-plex and 100 ng DNA, replicate 7	SAMN53262601
SRR36067977	Campylobacter coli, DBT kit, 36-plex and 100 ng DNA, replicate 8	SAMN53262601
SRR36067976	Campylobacter coli, DBT kit, 36-plex and 100 ng DNA, replicate 9	SAMN53262601
SRR36067975	Escherchia coli, DBT kit, 36-plex and 100 ng DNA, replicate 1	SAMN53262602
SRR36068008	Escherchia coli, DBT kit, 36-plex and 100 ng DNA, replicate 2	SAMN53262602
SRR36068007	Escherchia coli, DBT kit, 36-plex and 100 ng DNA, replicate 3	SAMN53262602
SRR36068006	Escherchia coli, DBT kit, 36-plex and 100 ng DNA, replicate 4	SAMN53262602
SRR36068005	Escherchia coli, DBT kit, 36-plex and 100 ng DNA, replicate 5	SAMN53262602
SRR36068004	Escherchia coli, DBT kit, 36-plex and 100 ng DNA, replicate 6	SAMN53262602
SRR36068003	Escherchia coli, DBT kit, 36-plex and 100 ng DNA, replicate 7	SAMN53262602
SRR36068002	Escherchia coli, DBT kit, 36-plex and 100 ng DNA, replicate 8	SAMN53262602
SRR36068001	Escherchia coli, DBT kit, 36-plex and 100 ng DNA, replicate 9	SAMN53262602
SRR36068000	Enterococcus faecalis, DBT kit, 36-plex and 100 ng DNA, replicate 1	SAMN53262603
SRR36067999	Enterococcus faecalis, DBT kit, 36-plex and 100 ng DNA, replicate 2	SAMN53262603
SRR36067997	Enterococcus faecalis, DBT kit, 36-plex and 100 ng DNA, replicate 3	SAMN53262603
SRR36067996	Enterococcus faecalis, DBT kit, 36-plex and 100 ng DNA, replicate 4	SAMN53262603
SRR36067995	Enterococcus faecalis, DBT kit, 36-plex and 100 ng DNA, replicate 5	SAMN53262603
SRR36067994	Enterococcus faecalis, DBT kit, 36-plex and 100 ng DNA, replicate 6	SAMN53262603
SRR36067993	Enterococcus faecalis, DBT kit, 36-plex and 100 ng DNA, replicate 7	SAMN53262603
SRR36067992	Enterococcus faecalis, DBT kit, 36-plex and 100 ng DNA, replicate 8	SAMN53262603
SRR36067991	Enterococcus faecalis, DBT kit, 36-plex and 100 ng DNA, replicate 9	SAMN53262603
SRR36067990	Staphylococcus faecalis, DBT kit, 36-plex and 100 ng DNA, replicate 1	SAMN53262604
SRR36067989	Staphylococcus faecalis, DBT kit, 36-plex and 100 ng DNA, replicate 2	SAMN53262604
SRR36067988	Staphylococcus faecalis, DBT kit, 36-plex and 100 ng DNA, replicate 3	SAMN53262604
SRR36067986	Staphylococcus faecalis, DBT kit, 36-plex and 100 ng DNA, replicate 4	SAMN53262604
SRR36067985	Staphylococcus faecalis, DBT kit, 36-plex and 100 ng DNA, replicate 5	SAMN53262604
SRR36067984	Staphylococcus faecalis, DBT kit, 36-plex and 100 ng DNA, replicate 6	SAMN53262604
SRR36067983	Staphylococcus faecalis, DBT kit, 36-plex and 100 ng DNA, replicate 7	SAMN53262604
SRR36067982	Staphylococcus faecalis, DBT kit, 36-plex and 100 ng DNA, replicate 8	SAMN53262604
SRR36067981	Staphylococcus faecalis, DBT kit, 36-plex and 100 ng DNA, replicate 9	SAMN53262604
SRR36035660	Campylobacter coli, DBT kit, 12-plex and 200 ng DNA, replicate 1	SAMN53262601
SRR36035659	Campylobacter coli, DBT kit, 12-plex and 200 ng DNA, replicate 2	SAMN53262601
SRR36035656	Campylobacter coli, DBT kit, 12-plex and 200 ng DNA, replicate 3	SAMN53262601
SRR36035655	Escherchia coli, DBT kit, 12-plex and 200 ng DNA, replicate 1	SAMN53262602

SRR36035654	Escherchia coli, DBT kit, 12-plex and 200 ng DNA, replicate 2	SAMN53262602
SRR36035653	Escherchia coli, DBT kit, 12-plex and 200 ng DNA, replicate 3	SAMN53262602
SRR36035652	Enterococcus faecalis, DBT kit, 12-plex and 200 ng DNA, replicate 1	SAMN53262603
SRR36035651	Enterococcus faecalis, DBT kit, 12-plex and 200 ng DNA, replicate 2	SAMN53262603
SRR36035650	Enterococcus faecalis, DBT kit, 12-plex and 200 ng DNA, replicate 3	SAMN53262603
SRR36035649	Staphylococcus faecalis, DBT kit, 12-plex and 200 ng DNA, replicate 1	SAMN53262604
SRR36035658	Staphylococcus faecalis, DBT kit, 12-plex and 200 ng DNA, replicate 2	SAMN53262604
SRR36035657	Staphylococcus faecalis, DBT kit, 12-plex and 200 ng DNA, replicate 3	SAMN53262604
SRR36068094	Campylobacter coli, DBT kit, 24-plex and 200 ng DNA, replicate 1	SAMN53262601
SRR36068093	Campylobacter coli, DBT kit, 24-plex and 200 ng DNA, replicate 2	SAMN53262601
SRR36068082	Campylobacter coli, DBT kit, 24-plex and 200 ng DNA, replicate 3	SAMN53262601
SRR36068077	Campylobacter coli, DBT kit, 24-plex and 200 ng DNA, replicate 4	SAMN53262601
SRR36068076	Campylobacter coli, DBT kit, 24-plex and 200 ng DNA, replicate 5	SAMN53262601
SRR36068075	Campylobacter coli, DBT kit, 24-plex and 200 ng DNA, replicate 6	SAMN53262601
SRR36068074	Escherchia coli, DBT kit, 24-plex and 200 ng DNA, replicate 1	SAMN53262602
SRR36068073	Escherchia coli, DBT kit, 24-plex and 200 ng DNA, replicate 2	SAMN53262602
SRR36068072	Escherchia coli, DBT kit, 24-plex and 200 ng DNA, replicate 3	SAMN53262602
SRR36068071	Escherchia coli, DBT kit, 24-plex and 200 ng DNA, replicate 4	SAMN53262602
SRR36068092	Escherchia coli, DBT kit, 24-plex and 200 ng DNA, replicate 5	SAMN53262602
SRR36068091	Escherchia coli, DBT kit, 24-plex and 200 ng DNA, replicate 6	SAMN53262602
SRR36068090	Enterococcus faecalis, DBT kit, 24-plex and 200 ng DNA, replicate 1	SAMN53262603
SRR36068089	Enterococcus faecalis, DBT kit, 24-plex and 200 ng DNA, replicate 2	SAMN53262603
SRR36068088	Enterococcus faecalis, DBT kit, 24-plex and 200 ng DNA, replicate 3	SAMN53262603
SRR36068087	Enterococcus faecalis, DBT kit, 24-plex and 200 ng DNA, replicate 4	SAMN53262603
SRR36068086	Enterococcus faecalis, DBT kit, 24-plex and 200 ng DNA, replicate 5	SAMN53262603
SRR36068085	Enterococcus faecalis, DBT kit, 24-plex and 200 ng DNA, replicate 6	SAMN53262603
SRR36068084	Staphylococcus faecalis, DBT kit, 24-plex and 200 ng DNA, replicate 1	SAMN53262604
SRR36068083	Staphylococcus faecalis, DBT kit, 24-plex and 200 ng DNA, replicate 2	SAMN53262604
SRR36068081	Staphylococcus faecalis, DBT kit, 24-plex and 200 ng DNA, replicate 3	SAMN53262604
SRR36068080	Staphylococcus faecalis, DBT kit, 24-plex and 200 ng DNA, replicate 4	SAMN53262604
SRR36068079	Staphylococcus faecalis, DBT kit, 24-plex and 200 ng DNA, replicate 5	SAMN53262604
SRR36068078	Staphylococcus faecalis, DBT kit, 24-plex and 200 ng DNA, replicate 6	SAMN53262604
SRR36067883	Campylobacter coli, DBT kit, 36-plex and 200 ng DNA, replicate 1	SAMN53262601
SRR36067882	Campylobacter coli, DBT kit, 36-plex and 200 ng DNA, replicate 2	SAMN53262601
SRR36067871	Campylobacter coli, DBT kit, 36-plex and 200 ng DNA, replicate 3	SAMN53262601
SRR36067860	Campylobacter coli, DBT kit, 36-plex and 200 ng DNA, replicate 4	SAMN53262601
SRR36067853	Campylobacter coli, DBT kit, 36-plex and 200 ng DNA, replicate 5	SAMN53262601
SRR36067852	Campylobacter coli, DBT kit, 36-plex and 200 ng DNA, replicate 6	SAMN53262601
SRR36067851	Campylobacter coli, DBT kit, 36-plex and 200 ng DNA, replicate 7	SAMN53262601
SRR36067850	Campylobacter coli, DBT kit, 36-plex and 200 ng DNA, replicate 8	SAMN53262601
SRR36067849	Campylobacter coli, DBT kit, 36-plex and 200 ng DNA, replicate 9	SAMN53262601
SRR36067848	Escherchia coli, DBT kit, 36-plex and 200 ng DNA, replicate 1	SAMN53262602
SRR36067881	Escherchia coli, DBT kit, 36-plex and 200 ng DNA, replicate 2	SAMN53262602

SRR36067880	Escherchia coli, DBT kit, 36-plex and 200 ng DNA, replicate 3	SAMN53262602
SRR36067879	Escherchia coli, DBT kit, 36-plex and 200 ng DNA, replicate 4	SAMN53262602
SRR36067878	Escherchia coli, DBT kit, 36-plex and 200 ng DNA, replicate 5	SAMN53262602
SRR36067877	Escherchia coli, DBT kit, 36-plex and 200 ng DNA, replicate 6	SAMN53262602
SRR36067876	Escherchia coli, DBT kit, 36-plex and 200 ng DNA, replicate 7	SAMN53262602
SRR36067875	Escherchia coli, DBT kit, 36-plex and 200 ng DNA, replicate 8	SAMN53262602
SRR36067874	Escherchia coli, DBT kit, 36-plex and 200 ng DNA, replicate 9	SAMN53262602
SRR36067873	Enterococcus faecalis, DBT kit, 36-plex and 200 ng DNA, replicate 1	SAMN53262603
SRR36067872	Enterococcus faecalis, DBT kit, 36-plex and 200 ng DNA, replicate 2	SAMN53262603
SRR36067870	Enterococcus faecalis, DBT kit, 36-plex and 200 ng DNA, replicate 3	SAMN53262603
SRR36067869	Enterococcus faecalis, DBT kit, 36-plex and 200 ng DNA, replicate 4	SAMN53262603
SRR36067868	Enterococcus faecalis, DBT kit, 36-plex and 200 ng DNA, replicate 5	SAMN53262603
SRR36067867	Enterococcus faecalis, DBT kit, 36-plex and 200 ng DNA, replicate 6	SAMN53262603
SRR36067866	Enterococcus faecalis, DBT kit, 36-plex and 200 ng DNA, replicate 7	SAMN53262603
SRR36067865	Enterococcus faecalis, DBT kit, 36-plex and 200 ng DNA, replicate 8	SAMN53262603
SRR36067864	Enterococcus faecalis, DBT kit, 36-plex and 200 ng DNA, replicate 9	SAMN53262603
SRR36067863	Staphylococcus faecalis, DBT kit, 36-plex and 200 ng DNA, replicate 1	SAMN53262604
SRR36067862	Staphylococcus faecalis, DBT kit, 36-plex and 200 ng DNA, replicate 2	SAMN53262604
SRR36067861	Staphylococcus faecalis, DBT kit, 36-plex and 200 ng DNA, replicate 3	SAMN53262604
SRR36067859	Staphylococcus faecalis, DBT kit, 36-plex and 200 ng DNA, replicate 4	SAMN53262604
SRR36067858	Staphylococcus faecalis, DBT kit, 36-plex and 200 ng DNA, replicate 5	SAMN53262604
SRR36067857	Staphylococcus faecalis, DBT kit, 36-plex and 200 ng DNA, replicate 6	SAMN53262604
SRR36067856	Staphylococcus faecalis, DBT kit, 36-plex and 200 ng DNA, replicate 7	SAMN53262604
SRR36067855	Staphylococcus faecalis, DBT kit, 36-plex and 200 ng DNA, replicate 8	SAMN53262604
SRR36067854	Staphylococcus faecalis, DBT kit, 36-plex and 200 ng DNA, replicate 9	SAMN53262604
SRR36067973	Campylobacter coli, DBT kit, 12-plex and 50 ng DNA, replicate 1	SAMN53262601
SRR36067972	Campylobacter coli, DBT kit, 12-plex and 50 ng DNA, replicate 2	SAMN53262601
SRR36067969	Campylobacter coli, DBT kit, 12-plex and 50 ng DNA, replicate 3	SAMN53262601
SRR36067968	Escherchia coli, DBT kit, 12-plex and 50 ng DNA, replicate 1	SAMN53262602
SRR36067967	Escherchia coli, DBT kit, 12-plex and 50 ng DNA, replicate 2	SAMN53262602
SRR36067966	Escherchia coli, DBT kit, 12-plex and 50 ng DNA, replicate 3	SAMN53262602
SRR36067965	Enterococcus faecalis, DBT kit, 12-plex and 50 ng DNA, replicate 1	SAMN53262603
SRR36067964	Enterococcus faecalis, DBT kit, 12-plex and 50 ng DNA, replicate 2	SAMN53262603
SRR36067963	Enterococcus faecalis, DBT kit, 12-plex and 50 ng DNA, replicate 3	SAMN53262603
SRR36067962	Staphylococcus faecalis, DBT kit, 12-plex and 50 ng DNA, replicate 1	SAMN53262604
SRR36067971	Staphylococcus faecalis, DBT kit, 12-plex and 50 ng DNA, replicate 2	SAMN53262604
SRR36067970	Staphylococcus faecalis, DBT kit, 12-plex and 50 ng DNA, replicate 3	SAMN53262604
SRR36068140	Campylobacter coli, DBT kit, 24-plex and 50 ng DNA, replicate 1	SAMN53262601
SRR36068139	Campylobacter coli, DBT kit, 24-plex and 50 ng DNA, replicate 2	SAMN53262601
SRR36068128	Campylobacter coli, DBT kit, 24-plex and 50 ng DNA, replicate 3	SAMN53262601
SRR36068123	Campylobacter coli, DBT kit, 24-plex and 50 ng DNA, replicate 4	SAMN53262601
SRR36068122	Campylobacter coli, DBT kit, 24-plex and 50 ng DNA, replicate 5	SAMN53262601

SRR36068121	Campylobacter coli, DBT kit, 24-plex and 50 ng DNA, replicate 6	SAMN53262601
SRR36068120	Escherchia coli, DBT kit, 24-plex and 50 ng DNA, replicate 1	SAMN53262602
SRR36068119	Escherchia coli, DBT kit, 24-plex and 50 ng DNA, replicate 2	SAMN53262602
SRR36068118	Escherchia coli, DBT kit, 24-plex and 50 ng DNA, replicate 3	SAMN53262602
SRR36068117	Escherchia coli, DBT kit, 24-plex and 50 ng DNA, replicate 4	SAMN53262602
SRR36068138	Escherchia coli, DBT kit, 24-plex and 50 ng DNA, replicate 5	SAMN53262602
SRR36068137	Escherchia coli, DBT kit, 24-plex and 50 ng DNA, replicate 6	SAMN53262602
SRR36068136	Enterococcus faecalis, DBT kit, 24-plex and 50 ng DNA, replicate 1	SAMN53262603
SRR36068135	Enterococcus faecalis, DBT kit, 24-plex and 50 ng DNA, replicate 2	SAMN53262603
SRR36068134	Enterococcus faecalis, DBT kit, 24-plex and 50 ng DNA, replicate 3	SAMN53262603
SRR36068133	Enterococcus faecalis, DBT kit, 24-plex and 50 ng DNA, replicate 4	SAMN53262603
SRR36068132	Enterococcus faecalis, DBT kit, 24-plex and 50 ng DNA, replicate 5	SAMN53262603
SRR36068131	Enterococcus faecalis, DBT kit, 24-plex and 50 ng DNA, replicate 6	SAMN53262603
SRR36068130	Staphylococcus faecalis, DBT kit, 24-plex and 50 ng DNA, replicate 1	SAMN53262604
SRR36068129	Staphylococcus faecalis, DBT kit, 24-plex and 50 ng DNA, replicate 2	SAMN53262604
SRR36068127	Staphylococcus faecalis, DBT kit, 24-plex and 50 ng DNA, replicate 3	SAMN53262604
SRR36068126	Staphylococcus faecalis, DBT kit, 24-plex and 50 ng DNA, replicate 4	SAMN53262604
SRR36068125	Staphylococcus faecalis, DBT kit, 24-plex and 50 ng DNA, replicate 5	SAMN53262604
SRR36068124	Staphylococcus faecalis, DBT kit, 24-plex and 50 ng DNA, replicate 6	SAMN53262604
SRR36068624	Campylobacter coli, DBT kit, 36-plex and 50 ng DNA, replicate 1	SAMN53262601
SRR36068623	Campylobacter coli, DBT kit, 36-plex and 50 ng DNA, replicate 2	SAMN53262601
SRR36068612	Campylobacter coli, DBT kit, 36-plex and 50 ng DNA, replicate 3	SAMN53262601
SRR36068601	Campylobacter coli, DBT kit, 36-plex and 50 ng DNA, replicate 4	SAMN53262601
SRR36068594	Campylobacter coli, DBT kit, 36-plex and 50 ng DNA, replicate 5	SAMN53262601
SRR36068593	Campylobacter coli, DBT kit, 36-plex and 50 ng DNA, replicate 6	SAMN53262601
SRR36068592	Campylobacter coli, DBT kit, 36-plex and 50 ng DNA, replicate 7	SAMN53262601
SRR36068591	Campylobacter coli, DBT kit, 36-plex and 50 ng DNA, replicate 8	SAMN53262601
SRR36068590	Campylobacter coli, DBT kit, 36-plex and 50 ng DNA, replicate 9	SAMN53262601
SRR36068589	Escherchia coli, DBT kit, 36-plex and 50 ng DNA, replicate 1	SAMN53262602
SRR36068622	Escherchia coli, DBT kit, 36-plex and 50 ng DNA, replicate 2	SAMN53262602
SRR36068621	Escherchia coli, DBT kit, 36-plex and 50 ng DNA, replicate 3	SAMN53262602
SRR36068620	Escherchia coli, DBT kit, 36-plex and 50 ng DNA, replicate 4	SAMN53262602
SRR36068619	Escherchia coli, DBT kit, 36-plex and 50 ng DNA, replicate 5	SAMN53262602
SRR36068618	Escherchia coli, DBT kit, 36-plex and 50 ng DNA, replicate 6	SAMN53262602
SRR36068617	Escherchia coli, DBT kit, 36-plex and 50 ng DNA, replicate 7	SAMN53262602
SRR36068616	Escherchia coli, DBT kit, 36-plex and 50 ng DNA, replicate 8	SAMN53262602
SRR36068615	Escherchia coli, DBT kit, 36-plex and 50 ng DNA, replicate 9	SAMN53262602
SRR36068614	Enterococcus faecalis, DBT kit, 36-plex and 50 ng DNA, replicate 1	SAMN53262603
SRR36068613	Enterococcus faecalis, DBT kit, 36-plex and 50 ng DNA, replicate 2	SAMN53262603
SRR36068611	Enterococcus faecalis, DBT kit, 36-plex and 50 ng DNA, replicate 3	SAMN53262603
SRR36068610	Enterococcus faecalis, DBT kit, 36-plex and 50 ng DNA, replicate 4	SAMN53262603
SRR36068609	Enterococcus faecalis, DBT kit, 36-plex and 50 ng DNA, replicate 5	SAMN53262603
SRR36068608	Enterococcus faecalis, DBT kit, 36-plex and 50 ng DNA, replicate 6	SAMN53262603

SRR36068607	Enterococcus faecalis, DBT kit, 36-plex and 50 ng DNA, replicate 7	SAMN53262603
SRR36068606	Enterococcus faecalis, DBT kit, 36-plex and 50 ng DNA, replicate 8	SAMN53262603
SRR36068605	Enterococcus faecalis, DBT kit, 36-plex and 50 ng DNA, replicate 9	SAMN53262603
SRR36068604	Staphylococcus faecalis, DBT kit, 36-plex and 50 ng DNA, replicate 1	SAMN53262604
SRR36068603	Staphylococcus faecalis, DBT kit, 36-plex and 50 ng DNA, replicate 2	SAMN53262604
SRR36068602	Staphylococcus faecalis, DBT kit, 36-plex and 50 ng DNA, replicate 3	SAMN53262604
SRR36068600	Staphylococcus faecalis, DBT kit, 36-plex and 50 ng DNA, replicate 4	SAMN53262604
SRR36068599	Staphylococcus faecalis, DBT kit, 36-plex and 50 ng DNA, replicate 5	SAMN53262604
SRR36068598	Staphylococcus faecalis, DBT kit, 36-plex and 50 ng DNA, replicate 6	SAMN53262604
SRR36068597	Staphylococcus faecalis, DBT kit, 36-plex and 50 ng DNA, replicate 7	SAMN53262604
SRR36068596	Staphylococcus faecalis, DBT kit, 36-plex and 50 ng DNA, replicate 8	SAMN53262604
SRR36068595	Staphylococcus faecalis, DBT kit, 36-plex and 50 ng DNA, replicate 9	SAMN53262604

Supplementary Table S2: Firth's bias-reduced logistic regression of sequencing success and detection outcomes across multiplexing and DNA input configurations.

Outcome	Contrast	Odds ratio	CI	P value
mean.cov.30x	200 ng vs 100 ng	0.2533640612662302	0.02 - 2.52	0.2460831981313264
mean.cov.30x	24-plex vs 12-plex	0.3555708995088734	0.03 - 2.74	0.3328450908618872
mean.cov.30x	24-plex × 200 ng	0.13464850681727122	0.01 - 2.82	0.18494886801426103
mean.cov.30x	24-plex × 50 ng	0.37061440942536844	0 - 13.29	0.5976942943832776
mean.cov.30x	36-plex vs 12-plex	0.15411335985739194	0.01 - 1.01	0.05155194477982361
mean.cov.30x	36-plex × 200 ng	0.02245660758092578	0 - 0.58	0.023723894174447757
mean.cov.30x	36-plex × 50 ng	0.10031954724176445	0 - 2.97	0.18494988574487758
mean.cov.30x	50 ng vs 100 ng	3.7630680084310306	0.15 - 614.95	0.42500160568426926
mlst	200 ng vs 100 ng	0.1277589113728279	0 - 2.21	0.16804126577965106
mlst	24-plex vs 12-plex	1.9145513630570594	0.01 - 380.22	0.7556863124186071
mlst	24-plex × 200 ng	0.07569143828878788	0 - 18.89	0.2872817406398205
mlst	24-plex × 50 ng	1.0000000000000062	0 - 732.13	1
mlst	36-plex vs 12-plex	0.07278994597489083	0 - 0.8	0.02954105789856709
mlst	36-plex × 200 ng	1.2772612190156387	0.05 - 217.38	0.8905765165315199
mlst	36-plex × 50 ng	4.483710358589998	0.02 - 1055.72	0.5106138936825596
mlst	50 ng vs 100 ng	0.9999999999999953	0 - 203.29	1
res	200 ng vs 100 ng	0.2884087074702111	0 - 6.43	0.440291465960011
res	24-plex vs 12-plex	1.9271382266992791	0.01 - 374.09	0.750896722604488
res	24-plex × 200 ng	0.5991865082998489	0 - 158.8	0.8274017605973527
res	24-plex × 50 ng	1.0000000000000084	0 - 702.19	1
res	36-plex vs 12-plex	0.35201637190565416	0 - 4.33	0.46184192888973763
res	36-plex × 200 ng	0.4051012304973266	0.01 - 71.72	0.6442011626939089
res	36-plex × 50 ng	8.104405622804402	0.03 - 4180.68	0.4172496526605042
res	50 ng vs 100 ng	0.9999999999999911	0.01 - 197.34	1

success	200 ng vs 100 ng	0.2533640612662302	0.02 - 2.52	0.2460831981313264
success	24-plex vs 12-plex	0.3555708995088734	0.03 - 2.74	0.3328450908618872
success	24-plex × 200 ng	0.13464850681727122	0.01 - 2.82	0.18494886801426103
success	24-plex × 50 ng	0.37061440942536844	0 - 13.29	0.5976942943832776
success	36-plex vs 12-plex	0.15411335985739194	0.01 - 1.01	0.05155194477982361
success	36-plex × 200 ng	0.02245660758092578	0 - 0.58	0.023723894174447757
success	36-plex × 50 ng	0.10031954724176445	0 - 2.97	0.18494988574487758
success	50 ng vs 100 ng	3.7630680084310306	0.15 - 614.95	0.42500160568426926
Firth's penalized likelihood logistic models were fitted for each binary outcome—30× genome coverage (mean.cov.30x), MLST detection (mlst), AMR gene detection (res), and overall sequencing success (success)—to evaluate the effect of multiplexing level and input DNA concentration, including interaction terms. Odds ratios (ORs) represent the relative odds of successful outcome for each contrast compared with the designated reference category (12-plex, 100 ng input). 95 % confidence intervals (CIs) and Wald-type p-values are shown. Values < 1 indicate lower odds and > 1 indicate higher odds of success relative to the reference configuration.				

Table S3: Effect of multiplexing level and input DNA on sequencing outcome.

**Strain**	**Multiplex level**	**Input DNA concentration (ng)**	**Cov 30x (n/N, %)**	**MLST (n/N, %)**	**ARG (n/N, %)**	**Overall success (n/N, %)**
C. coli	12	100	2/3 (66.67%)	3/3 (100.00%)	3/3 (100.00%)	2/3 (66.67%)
E. faecalis	12	100	3/3 (100.00%)	3/3 (100.00%)	3/3 (100.00%)	3/3 (100.00%)
E. coli	12	100	3/3 (100.00%)	3/3 (100.00%)	3/3 (100.00%)	3/3 (100.00%)
S. hominis	12	100	3/3 (100.00%)	3/3 (100.00%)	3/3 (100.00%)	3/3 (100.00%)
C. coli	12	200	1/3 (33.33%)	2/3 (66.67%)	3/3 (100.00%)	1/3 (33.33%)
E. faecalis	12	200	3/3 (100.00%)	3/3 (100.00%)	3/3 (100.00%)	3/3 (100.00%)
E. coli	12	200	2/3 (66.67%)	2/3 (66.67%)	2/3 (66.67%)	2/3 (66.67%)
S. hominis	12	200	3/3 (100.00%)	3/3 (100.00%)	3/3 (100.00%)	3/3 (100.00%)
C. coli	12	50	3/3 (100.00%)	3/3 (100.00%)	3/3 (100.00%)	3/3 (100.00%)
E. faecalis	12	50	3/3 (100.00%)	3/3 (100.00%)	3/3 (100.00%)	3/3 (100.00%)
E. coli	12	50	3/3 (100.00%)	3/3 (100.00%)	3/3 (100.00%)	3/3 (100.00%)
S. hominis	12	50	3/3 (100.00%)	3/3 (100.00%)	3/3 (100.00%)	3/3 (100.00%)
C. coli	24	100	0/6 (0.00%)	2/6 (33.33%)	5/6 (83.33%)	0/6 (0.00%)
E. faecalis	24	100	6/6 (100.00%)	6/6 (100.00%)	6/6 (100.00%)	6/6 (100.00%)
E. coli	24	100	5/6 (83.33%)	6/6 (100.00%)	6/6 (100.00%)	5/6 (83.33%)
S. hominis	24	100	6/6 (100.00%)	6/6 (100.00%)	6/6 (100.00%)	6/6 (100.00%)
C. coli	24	200	0/6 (0.00%)	1/6 (16.67%)	4/6 (66.67%)	0/6 (0.00%)
E. faecalis	24	200	6/6 (100.00%)	6/6 (100.00%)	6/6 (100.00%)	6/6 (100.00%)
E. coli	24	200	0/6 (0.00%)	5/6 (83.33%)	6/6 (100.00%)	0/6 (0.00%)
S. hominis	24	200	0/6 (0.00%)	1/6 (16.67%)	6/6 (100.00%)	0/6 (0.00%)
C. coli	24	50	2/6 (33.33%)	6/6 (100.00%)	6/6 (100.00%)	2/6 (33.33%)

E. faecalis	24	50	6/6 (100.00%)	6/6 (100.00%)	6/6 (100.00%)	6/6 (100.00%)
E. coli	24	50	6/6 (100.00%)	6/6 (100.00%)	6/6 (100.00%)	6/6 (100.00%)
S. hominis	24	50	6/6 (100.00%)	6/6 (100.00%)	6/6 (100.00%)	6/6 (100.00%)
C. coli	36	100	0/9 (0.00%)	1/9 (11.11%)	8/9 (88.89%)	0/9 (0.00%)
E. faecalis	36	100	9/9 (100.00%)	9/9 (100.00%)	9/9 (100.00%)	9/9 (100.00%)
E. coli	36	100	6/9 (66.67%)	8/9 (88.89%)	7/9 (77.78%)	6/9 (66.67%)
S. hominis	36	100	9/9 (100.00%)	9/9 (100.00%)	9/9 (100.00%)	9/9 (100.00%)
C. coli	36	200	0/9 (0.00%)	0/9 (0.00%)	2/9 (22.22%)	0/9 (0.00%)
E. faecalis	36	200	0/9 (0.00%)	8/9 (88.89%)	9/9 (100.00%)	0/9 (0.00%)
E. coli	36	200	1/9 (11.11%)	7/9 (77.78%)	9/9 (100.00%)	1/9 (11.11%)
S. hominis	36	200	0/9 (0.00%)	2/9 (22.22%)	2/9 (22.22%)	0/9 (0.00%)
C. coli	36	50	6/9 (66.67%)	9/9 (100.00%)	9/9 (100.00%)	6/9 (66.67%)
E. faecalis	36	50	7/9 (77.78%)	9/9 (100.00%)	9/9 (100.00%)	7/9 (77.78%)
E. coli	36	50	4/9 (44.44%)	8/9 (88.89%)	9/9 (100.00%)	4/9 (44.44%)
S. hominis	36	50	1/9 (11.11%)	7/9 (77.78%)	9/9 (100.00%)	1/9 (11.11%)

Strains included in the study: *Escherichia coli* (GCA_029094485), *Campylobacter coli* (GCA_949361535), *Enterococcus faecalis* (GCA_029167565), and *Staphylococcus hominis* (JBRFSD000000000); multiplexing level (*n* samples); input DNA (ng); proportion of samples with successful binary outcomes (*n/N, %*) for coverage ≥30×, full MLST, detection of all ARG, and overall WGS success, defined as meeting all three criteria.

Supplementary Figures

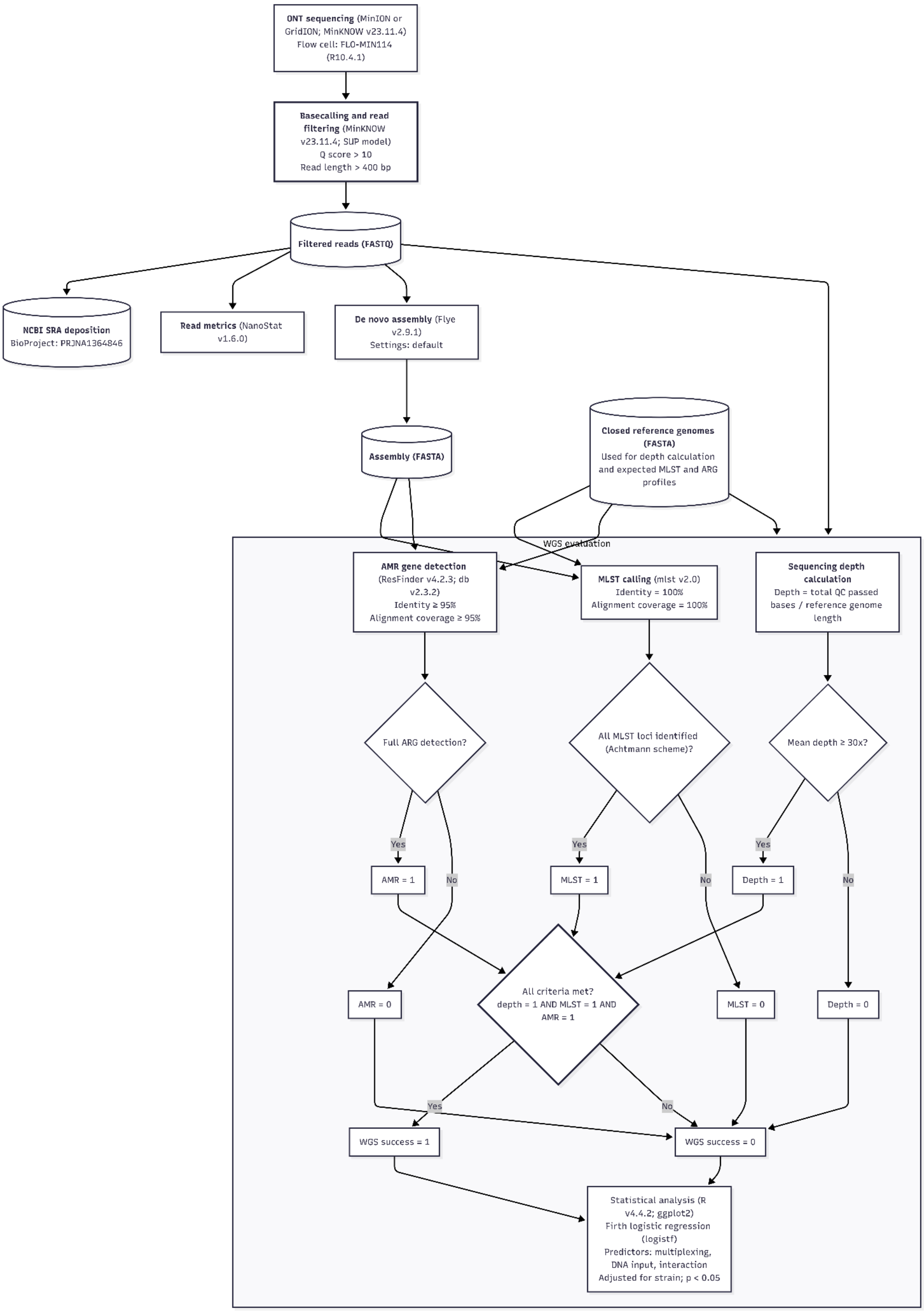
ONT sequencing (MinION or GridION; MinKNOW v23.11.4) Flow cell: FLO-MIN114 (R10.4.1)
Basecalling and read filtering (MinKNOW v23.11.4; SUP model) Q score > 10 Read length > 400 bp
Filtered reads (FASTQ)
NCBI SRA deposition BioProject: PRJNA1364846
Read metrics (NanoStat v1.6.0)
De novo assembly (Flye v2.9.1) Settings: default
Assembly (FASTA)
Closed reference genomes (FASTA) Used for depth calculation and expected MLST and ARG profiles
WGS evaluation
AMR gene detection (ResFinder v4.2.3; db v2.3.2) Identity ≥ 95% Alignment coverage ≥ 95%
MLST calling (mlst v2.0) Identity = 100% Alignment coverage = 100%
Sequencing depth calculation Depth = total QC passed bases / reference genome length
Full ARG detection?
All MLST loci identified (Achtmann scheme)?
Mean depth ≥ 30x?
Yes
No
AMR = 1
MLST = 1
Depth = 1
AMR = 0
MLST = 0
Depth = 0
All criteria met? depth = 1 AND MLST = 1 AND AMR = 1
WGS success = 1
WGS success = 0
Statistical analysis (R v4.4.2; ggplot2) Firth logistic regression (logistf) Predictors: multiplexing, DNA input, interaction Adjusted for strain; p < 0.05

Supplementary Figure 1

Workflow and binary definition of WGS success. Sequencing was performed on MinION or GridION using R10.4.1 flow cells and MinKNOW v23.11.4, with basecalling and read filtering using the SUP model (Q-score >10; read length >400 bp). Filtered reads were assessed with NanoStat v1.6.0, assembled using Flye v2.9.1, and deposited in NCBI SRA (BioProject PRJNA1364846). Closed reference genomes were used to estimate sequencing depth (total QC-passed bases/reference genome length; ≥30× pass) and to define expected MLST and antimicrobial resistance gene profiles. MLST completeness was assessed using mlst v2.0 (all loci for the species-specific scheme), and ARG detection using ResFinder v4.2.3 (identity and coverage ≥95%). WGS success = 1 only when depth, MLST, and ARG criteria were all met; otherwise, 0.
